# Screening for celiac disease in poorly controlled type 2 diabetes mellitus: worth it or not?

**DOI:** 10.1186/s12902-017-0212-4

**Published:** 2017-10-06

**Authors:** Muhammed Kizilgul, Ozgur Ozcelik, Selvihan Beysel, Hakan Akinci, Seyfullah Kan, Bekir Ucan, Mahmut Apaydin, Erman Cakal

**Affiliations:** 1 Department of Endocrinology and Metabolism, Diskapi Training and Research Hospital, Ankara, Turkey; 2 Schulze Diabetes Institute, University of Minnesota, Minneapolis, MN USA; 3 Department of Gastroenterology, Diskapi Training and Research Hospital, Ankara, Turkey

**Keywords:** Type 2 diabetes mellitus, Celiac disease, Tissue transglutaminase antibody

## Abstract

**Background:**

Recent studies have demonstrated that immune factors might have a role in the pathophysiology of insulin resistance and type 2 diabetes mellitus (T2DM). Inappropriate glycemic control in patients with T2DM is an important risk factor for the occurrence of diabetes complications. The prevalence of celiac disease (CD) is high in type 1 diabetes mellitus however, there are scarce data about its prevalence in T2DM. Our aim was to investigate the prevalence of celiac disease among insulin-using type 2 diabetes patients with inappropriate glycemic control.

**Methods:**

IgA tissue transglutaminase antibodies (tTGA IgA) test was performed as a screening test. A total of 135 patients with T2DM whose control of glycemia is inappropriate (HbAlc value >7%) in spite of using insulin treatment for at least 3-months (only insulin or insulin with oral antidiabetic drugs) and 115 healthy controls were enrolled in the study. Upper gastrointestinal endoscopy with duodenal biopsy was performed to all patients with raised tTGA IgA or selective lgA deficiency.

**Results:**

Gender, age, body mass index (BMI) and tTGA IgA, kreatinin, calcium, LDL-cholesterol (LDL-C), total cholesterol, 25-OH vitamin D_3_ levels were similar between groups. Systolic and diastolic blood pressure, waist circumference, fasting plasma glucose, postprandial plasma glucose, urea, sodium, HbA1c, LDL-C, triglyceride, vitamin B12 levels were significantly higher in DM group (*p* < 0.0001). BMI, high-sensitive CRP, microalbuminuria, and AST, ALT, potassium, phosphorus levels were significantly higher in the T2DM group (*p* < 0.05). HDL-cholesterol and parathormone levels were significantly lower in the T2DM group (*p* < 0.05). Two of the 135 patients with T2DM were diagnosed with CD (1.45%).

**Conclusions:**

The prevalence of celiac disease among patients with type 2 diabetes, with poor glycemic control despite insulin therapy, is slightly higher than the actual CD prevalence in general population. Type 2 diabetic patients with inappropriate control of glycemia in spite of insulin treatment might be additionally tested for Celiac disease especially if they have low C-peptide levels.

## Background

Type 2 diabetes mellitus (T2DM), a chronic metabolic disorder, is an important community health problem in which prevalence has been increasing steadily all over the world [1]. Celiac disease (CD), a chronic autoimmune disease, is described as small intestinal inflammation and villous atrophy (VA) induced by gluten exposure in genetically susceptible people [2] and its prevalence approximately reaches 1/100 in western European countries [3]. A recent meta-analysis demonstrated that more than 1 in 20 patients with type 1 diabetes (T1DM) had celiac disease confirmed by biopsy [4]. Serology has a key role in the diagnosis of CD. Evaluating IgA tissue transglutaminase antibody (tTGA) is now considered as the best strategy for CD serological screening since it has the highest sensitivity in diagnosing of CD (up to % 97) [5]. The impaired beta-cell function is accepted as the main physiopathological mechanism for overt type 2 diabetes; however, the leading cause of this disease remains unknown [6]. Recent studies have demonstrated that both innate immune and adaptive immune system might have a role in the pathophysiology of insulin resistance and T2DM [7]. Insulin resistance and progressive loss of insulin secretion capacity due to either beta-cell dysfunction and/or beta-cell loss are main characteristics of T2DM [8]. High-fat diet related secretion of proinflammatory cytokines [9], endoplasmic reticulum stress via activation of toll-like receptors [10–12], activation of NLRP3 inflammasome upregulation [13] and promotion of glucose homeostasis by TNF-α blocker [14], the association of insulin resistance and dietary fatty acids with activation of B and T lymphocytes [15–17] are all evidence which may suggest the role of autoimmunity in the etiopathogenesis of T2DM. Few studies have investigated the prevalence of CD in patients with T2DM [18–20]. However, only one study investigated tTGA prevalence in patients with T2DM, which demonstrated tTGA positivity in 11% of patients [21]. It is well-known that good glycemic control in diabetes decreases both the microvascular and macrovascular complications [22] however, many patients still have poor glycemic control [23]. Inappropriate glycemic control in patients with T2DM is an important risk factor for the occurrence of diabetes complications [24]. Patient and health care professional associated factors might have a role in poor glycemic control [25].

The purpose of this paper was to evaluate the prevalence of tTGA IgA positivity and possible celiac disease in T2DM patients with poor glycemic control (HbAlc value >7%) despite receiving insulin treatment.

## Methods

We studied the frequency of tTGA IgA in patients with T2DM (*n* = 135) whose control of glycemia is inappropriate (HbAlc value >7%) in spite of using insulin therapy for at least 3-months (only insulin or insulin with oral antidiabetic drugs) and healthy controls (*n* = 115) attending the endocrinology outpatient clinic of Diskapi Training and Research Hospital in Turkey. Ethical standards of the Helsinki Declaration were followed. Approval of ethical committee in Diskapi Training and Research Hospital (Date: 26.01.2015, Decree No:19/36) and written informed consent of participants were obtained before the study. All treatments that patients received are considered standard care for their condition.

All patients with raised tTGA IgA and those with selective IgA deficiency were invited for further assessment. A medical history and evidence of diabetic complications were noted, together with an evaluation of gastrointestinal symptoms using a questionnaire administered by a single observer. Upper gastrointestinal endoscopy with duodenal biopsy was offered to all patients with raised tTGA IgA or selective lgA deficiency. Endoscopy was performed under sedation, and multiple biopsies were obtained from the second part of the duodenum or beyond. All biopsies were examined histologically according to Marsh’s criteria [26] by a single observer at the Department of Pathology who was unaware of the tTGA IgA titer. Those patients whose biopsies showed total or severe partial villous atrophy were regarded as having celiac disease. Autoantibodies (Anti-islet cell antibody, anti-insulin antibody, and anti-GAD antibody) were assessed in the patients diagnosed with CD. Patients with CD received advice about a gluten-free diet. Supplemental iron, folic acid, and calcium were prescribed as appropriate. Clinical, hematological and biochemical assessments were carried out every three months to evaluate the response to withdrawal of gluten. Direct questioning monitored dietary compliance. Demographic data and medical history of all individuals including diabetes duration, treatment protocol were noted; anthropometric measurements were carried out. Weight, height, waist circumference (WC), hip circumference (HC) and systolic and diastolic blood pressure (BP) were measured. Dividing the weight by the height squared (kg/m2) was used for the measurement of the body mass index (BMI). WC was determined by measuring the narrowest point between the lowest rib and iliac crest at minimal normal respiration. A standard mercury sphygmomanometer was used for the measurement of the blood pressure (BP), with the patient at rest and in the sitting posture for at least 10 min. The average of two calculations was noted.

### Measurement of anti-tissue transglutaminase antibodies (tTGA)

Tissue transglutaminase antibodies were measured using a human tTG enzyme-linked immunosorbent assay (ELISA) kit (AESKULISA, Aesku.lab Diagnostka, Wendelsheim, Germany). The intra-assay and inter-assay coefficient of variation were both lower than 10%.

Serum IgA levels of CD patients and healthy controls were measured for the exclusion of IgA deficiency. IgA level of lower than 0.05 g/L, indicative of selective IgA deficiency, was not observed in any cases.

### Statistical analysis

Statistical analysis was performed using JMP 11.0.0 software (SAS Institute, Cary, NC). Variables are presented as mean ± standard deviation (SD) or median (min-max), percentage (%). Normality was tested by Kolmogorov-Smirnov and Shapiro-Wilk W test. A two-sample *t*-test or one-way ANOVA were used for comparison of numerical means. Mann-Whitney U test was used for continuous variables between diabetes with and without celiac disease. The Chi-square test or Fisher’s exact test was used for categorical variables. Statistical significance was defined as a *p* < 0.05.

## Results

One hundred thirty-five subjects with T2DM and 115 healthy individuals were enrolled in the study. All patients underwent a first screening step for CD using tTGA IgA test. 3 patients (2.2%) in DM group and 1 patient (0.9%) in control group had increased level of tTGA IgA. These patients underwent upper gastrointestinal endoscopy with duodenal biopsy and anti-endomysial antibodies test. Two of the patients in DM group was diagnosed with CD (1.45%) (Tables 1 and 2). Autoantibodies (anti-islet cell antibody, anti-insulin antibody, and anti-GAD antibody) were assessed in the patients diagnosed with celiac disease. Both patients had negative results for anti-GAD, anti-insulin, and anti-islet cell antibodies. Gender, age, BMI and tTGA IgA, kreatinin, calcium, LDL-cholesterol, total cholesterol, 25-OH vitamin D_3_ levels were similar between groups. SBP, DBP, WC and fasting plasma glucose (FPG), postprandial plasma glucose (PPPG), urea, sodium, HbA1c, LDL-C, TG, vitamin B12 levels were significantly higher in DM group (*p* < 0.0001). BMI, hsCRP, microalbuminuria and AST, ALT, potassium, phosphorus levels were significantly higher in DM group (*p* < 0.05). HDL-C and parathormone (PTH) levels were significantly lower in DM group (*p* < 0.05). Plasma tTGA IgA concentrations were positively correlated with hsCRP (r:0.2138, *p*:0.0285) and HbA1c (r:0.197, *p*:0.0221). Plasma tTGA IgA concentrations were negatively correlated with LDL-cholesterol (r:-0.197, *p*:0.0232) and total cholesterol (r:-0.193, *p*:0.0367) (Table 3).

**Table 1:** Demographic and clinical characteristics of patients

	Groups				
	DM (n:135)		Control (n:115)		
Gender	*n*	%	*n*	%	*p*
Female	90	67	82	71	0.430
Male	45	33	33	29	
Celiac Disease	2	1.45	0	0	0.501
	Mean	Std Dev	Mean	Std Dev	*p*
Age (yrs)	52.7	6.8	50.9	8.1	0.069
SBP (mmHg)	129.5	15.4	114.4	13.6	<0.0001
DBP (mmHg)	80.5	9.9	71.2	9.1	<0.0001
BMI (kg/m^2^)	32.4	12.3	28.0	4.7	0.0002
WC (cm)	102.3	13.0	93.0	11.3	<0.0001
Duration of Diabetes (yrs)	12.1	7.1	–	–	
Total Insulin Dose (u)	54.7	30	–	–

**Table 2:** Biochemical parameters of the study population

Groups					
	DM (n:135)		Control (n:115)		
	Mean	Std Dev	Mean	Std Dev	*p*
tTGA IgA (U/mL)	7.5	27.3	3.8	3.8	0.128
hsCRP	8.0	8.8	3.7	10.5	0.003
FPG	219.0	82.5	94.9	10.9	<0.0001
PBG	307.0	82.8	109.3	23.1	<0.0001
Urea	31.1	9.5	26.6	7.5	<0.0001
Kreatinin	0.9	0.2	0.9	0.1	0.255
AST	25.0	13.9	21.0	5.6	0.006
ALT	27.5	20.0	21.0	10.6	0.0013
Sodium	137.4	2.3	139.2	1.7	<0.0001
Potassium	4.6	0.4	4.5	0.4	0.0005
Calcium	9.6	0.4	9.6	0.4	0.756
Phosphorus	3.7	0.6	3.4	0.6	0.0003
HbA1c	10.0	1.8	5.5	0.3	<0.0001
LDL-C	142.7	34.1	141.5	37.6	0.858
HDL-C	43.4	9.6	49.5	10.2	<0.0001
Total-C	194.7	43.0	199.7	44.8	0.349
TG	215.3	153.3	136.0	76.7	<0.0001
Microalbuminuria	42.4	73.5	17.0	24.8	0.0178
25-OHVitD3	16.7	14.7	15.9	14.7	0.566
PTH	46.1	21.2	58.3	24.8	0.0006
Vitamin B12	303.7	210.5	207.6	82.2	<0.0001

**Table 3:** The correlation between tTGA levels and clinical, biochemical and hormonal parameters in DM group

Variable	Correlation coefficient	*p*-value
Age	−0.057	0.513
SBP	0.0094	0.9162
DBP	−0.0004	0.996
BMI	−0.0162	0.854
WC	0.0528	0.5603
Duration of DM	−0.0336	0.7025
Total insulin use	−0.0318	0.7288
Anti-TPO	−0.0107	0.9138
Anti-Tg	−0.0104	0.9183
hsCRP	0.2138	0.0285
FPG	−0.0382	0.6615
PPG	0.0428	0.689
Urea	0.1641	0.0698
Creatinine	0.0243	0.781
AST	−0.0776	0.4054
ALT	−0.0826	0.3467
HbA1c	0.1976	0.0221
LDL-Cholesterol	−0.1974	0.0232
HDL-Cholesterol	0.0858	0.3494
Total-Cholesterol	−0.1933	0.0367
TG	−0.0785	0.3712
C-peptide	−0.137	0.1739

The diabetic patients were divided into three groups according to the C-peptide concentrations. The cut-off point of 0.51 was assumed. The first group consists of 12 patients with c-peptide level (normal range:0.51–2.7 ng/mL) lower than 0.51 ng/mL, the second group consists of 22 patients with a c-peptide level between 0.51–1.0 ng/m L and third group consist of 66 patients with c-peptide level higher than 1.0 ng/mL. The mean c-peptide concentrations were significantly different between groups (0.24 ± 0.23, 0.78 ± 0.17, 2.17 ± 0.10, respectively, *p* < 0.0001). The mean tTGA concentrations between groups were significantly different between groups (36.44 ± 8.67, 5.14 ± 6.41, 4.56 ± 3.70, respectively, *p* < 0.0039). Age, SBP, DBP, BMI, WC, duration of diabetes, total insülin dose and hsCRP, FPG, PPG, HbA1c were similar between groups (*p* > 0.05) (Table 4).

**Table 4:** Demographic, biochemical and clinical characteristics of diabetic patients when they divided into 3 categories based on their c-peptide concentrations

	1st group (n:13)		2nd group (n:22)		3rd group (n:66)		
	Mean	SD	Mean	SD	Mean	SD	*p*-value
Age (yrs)	53.38	5.28	56.64	6.06	54.08	8.79	0.362
SBP (mmHg)	126.15	17.58	127.62	11.36	130.00	16.13	0.647
DBP (mmHg)	77.69	12.35	79.29	7.79	80.94	10.19	0.518
BMI (kg/m^2^)	28.54	4.43	29.86	4.07	34.45	16.47	0.210
WC (cm)	95.85	15.85	101.50	7.80	103.53	14.04	0.168
Duration of Diabetes (yrs)	14.77	9.79	13.82	6.10	11.02	6.86	0.108
Total Insulin Dose (u)	54.46	25.09	62.10	34.98	53.76	27.59	0.535
tTGA IgA (U/mL)	36.44	88.13	5.15	6.39	4.57	4.64	0.004
hsCRP	5.20	8.60	6.74	5.63	7.85	7.63	0.579
FPG	227.69	153.26	200.00	66.04	217.89	71.14	0.591
PBG	274.71	128.90	322.21	88.55	304.83	80.31	0.506
HbA1c	10.13	2.08	10.69	1.93	9.69	1.70	0.080
C-peptide	0.24	0.20	0.78	0.14	2.18	1.01	<.0001

The first group consists of patients with c-peptide level (normal range:0.51–2.7 ng/mL) lower than 0.51 ng/mL, the second group consists of patients with a c-peptide level between 0.51–1.0 ng/m L and third group consist of a c-peptide level higher than 1.0 ng/mL

## Discussion

Accumulating evidence have suggested that both innate immune and adaptive immune system might have a role in the etiopathogenesis of T2DM. Circulating autoantibodies observed in T2DM may be a sign of autoimmune involvement. Expression of several β-cell antigens such as anti-GAD was increased by hyperglycemia and anti-GAD is the most frequently determined antibodies in phenotypic T2DM [27]. IL-1β is known to cause both pancreatic β cells impairment and destruction during the type 1 diabetes mellitus development. In human β cells, high levels of glucose cause production and release of IL-1β, which leads to upregulation of Fas receptor, NF-κB activation, apoptosis and impaired function of the β cell [28, 29]. B cells in T2DM patients could not produce anti-inflammatory interleukin-10 (IL-10), is effective in the control of autoimmune conditions, as a response to TLR2, TLR4 or TLR9 stimulation [30]. Therefore, decrease in IL-10 secretion as a response to stimulation by lipopolysaccharide leads to higher risk for T2DM development in elderly subjects [31].

Eventually, all these inflammatory and autoimmune pathways may lead to β-cell apoptosis and a further decrease in insulin levels. In light of this information, we aimed to investigate whether tissue transglutaminases and consequently CD may have a role in the explanation of poorly controlled diabetes. In our study, CD prevalence was slightly higher in patients with poorly controlled diabetes than in healthy individuals.

Transglutaminase catalyzes the posttranslational modification of proteins via catalyzing calcium-dependent acyl transfer reaction [32]. The presence of transglutaminases has been demonstrated in several endocrine glands including pituitary gland, thymus genital tract of the male, human normal and pathologic adrenal tissues and pancreatic islet cells. tTGA may have effected the function β-cells [33]. Transglutaminases participate in the dynamic events that controlling exocytosis of single secretory granules in the β-cells [34]. The activity of transglutaminase has an essential role in the insulin release by glucose [35].

Since tTGA IgA is very sensitive and specific in diagnosing of CD, it is a preferred screening test for CD [36]. Studies demonstrated that up to 12% of patients with T1DM screen positive for tTGA [37] however, there were scarce data about tTGA IgA prevalence in T2DM. Sjöberg et al. analyzed the presence of gliadin antibodies (GA) and endomysial antibodies (EMA) in patients with non-insulin dependent diabetes mellitus (NIDDM). Small bowel biopsy was performed to all EMA-positive diabetic patients. 92 (12.3%) patients with NIDDM had IgA-GA positivity, 1 patient has EMA positivity and 2 patients with NIDDM (2/745) was diagnosed with CD [18]. Page et al. investigated the presence of lgA-AGA in 769 T1DM and 1020 T2DM patients, and 73 had a high lgA-AGA level. Forty-nine of 73 patients agreed to a duodenal biopsy, and 13 (10 T1DM, 3 T2DM) were diagnosed with CD. The overall prevalence of CD in T2DM adult patients was found to be 1:340 [19]. Tourniaire et al. evaluated the presence of plasma gliadin antibodies in 80 type 1 diabetics, 80 type 2 diabetics, 80 non-diabetics without known auto-immune disease. IgA antibodies were observed in 6 type 1 diabetics (7.5%), 8 type 2 diabetics (10%) and 6 non-diabetics (7.5%) and only one patient were diagnosed with CD [20]. Kanungo et al. studied the frequency of TGA-Ab in patients with T2DM. 23 of 216 (11%) with T2DM were positive for TGA-Ab [21]. Usai et al. investigated the presence of IgA antigliadin antibodies (AGA) in 29 patients with T2DM, and no high AGA level observed [38]. In another study, the presence of AGA, tTGA was investigated in 43 subjects with latent autoimmune diabetes of adult (LADA) and 99 with T2DM. 4 (4.0%) of T2DM and 2 (4.6%) of LADA were found to be tTGA positive. The authors concluded CD did not seem to be associated with pancreatic autoimmunity in T2DM. Biopsy confirmation was not performed in this particular study [39].

In our study, the prevalence of CD disease was slightly higher than the actual prevalence of CD in general population. Patients with lower c-peptide levels in DM group had higher levels of tTGA IgA. This finding might suggest that type 2 diabetic patients with lower c-peptide levels, which possibly have a more autoimmune component of the disease, could have more prevalence of celiac disease.

## Conclusions

The prevalence of celiac disease in type 2 diabetes patients with inappropriate control of glycemia in spite of the insulin therapy, is slightly higher than the actual CD prevalence in general population. Type 2 diabetic patients with poor control of glycemia in spite of insulin treatment might be additionally tested for Celiac disease if they have low C-peptide levels. However, further prospective population-based studies are required for confirming this thesis.

## Abbreviations

BMI: Body mass index
CD: Celiac disease
DBP: Diastolic blood pressure
EMA: Anti endomysium antibody
ERS: Endoplasmic reticulum stress
FPG: Fasting plasma glucose
HC: Hip circumference
HLD-C: HDL cholesterol
LDL-C: LDL-cholesterol
NIDDM: Non-insulin dependent diabetes mellitus
PPPG: Post-prandial plasma glucose
SBP: Systolic blood pressure
T2DM: Type 2 diabetes mellitus
TLR: Tool-like receptors
tTGA IgA: Tissue transglutaminase antibody IgA
VA: Villous atrophy
WC: Waist circumference
